# Human Neutrophil Elastase Degrades SPLUNC1 and Impairs Airway Epithelial Defense against Bacteria

**DOI:** 10.1371/journal.pone.0064689

**Published:** 2013-05-31

**Authors:** Di Jiang, Sally E. Wenzel, Qun Wu, Russell P. Bowler, Christina Schnell, Hong Wei Chu

**Affiliations:** 1 Department of Medicine, National Jewish Health, Denver, Colorado, United States of America; 2 Department of Medicine, University of Pittsburgh School of Medicine, Pittsburgh, Pennsylvania, United States of America; Louisiana State University, United States of America

## Abstract

**Background:**

Acute exacerbations of chronic obstructive pulmonary disease (AECOPD) are a significant cause of mortality of COPD patients, and pose a huge burden on healthcare. One of the major causes of AECOPD is airway bacterial (e.g. nontypeable *Haemophilus influenzae* [NTHi]) infection. However, the mechanisms underlying bacterial infections during AECOPD remain poorly understood. As neutrophilic inflammation including increased release of human neutrophil elastase (HNE) is a salient feature of AECOPD, we hypothesized that HNE impairs airway epithelial defense against NTHi by degrading airway epithelial host defense proteins such as short palate, lung, and nasal epithelium clone 1 (SPLUNC1).

**Methodology/Main Results:**

Recombinant human SPLUNC1 protein was incubated with HNE to confirm SPLUNC1 degradation by HNE. To determine if HNE-mediated impairment of host defense against NTHi was SPLUNC1-dependent, SPLUNC1 protein was added to HNE-treated primary normal human airway epithelial cells. The *in vivo* function of SPLUNC1 in NTHi defense was investigated by infecting SPLUNC1 knockout and wild-type mice intranasally with NTHi. We found that: (1) HNE directly increased NTHi load in human airway epithelial cells; (2) HNE degraded human SPLUNC1 protein; (3) Recombinant SPLUNC1 protein reduced NTHi levels in HNE-treated human airway epithelial cells; (4) NTHi levels in lungs of SPLUNC1 knockout mice were increased compared to wild-type mice; and (5) SPLUNC1 was reduced in lungs of COPD patients.

**Conclusions:**

Our findings suggest that SPLUNC1 degradation by neutrophil elastase may increase airway susceptibility to bacterial infections. SPLUNC1 therapy likely attenuates bacterial infections during AECOPD.

## Introduction

COPD is the 3^rd^ leading cause of death in the US [1], [2]. One of the major challenges in COPD healthcare is to prevent and treat acute exacerbations of COPD (AECOPD). AECOPD is mainly caused by respiratory bacterial (e.g., nontypeable *Haemophilus influenzae*, NTHi) and viral (e.g., rhinovirus) infections [3]. Recurrent respiratory infections in COPD patients suggest impaired lung defense mechanisms. Although various mechanisms have been proposed or investigated for respiratory infection-induced AECOPD, many research questions remain to be answered. Further defining mechanisms underlying AECOPD is necessary to discover more effective therapies.

COPD lung including airways is inflamed, especially during AECOPD. One of the prominent inflammatory cells in COPD lungs is neutrophils [4], [5]. During their activation, neutrophils release various mediators such as cytokines and proteases, which contribute to COPD pathogenesis including AECOPD. Among the proteases released from neutrophils, human neutrophil elastase (HNE) is particularly important. First, HNE levels are higher in COPD patients than normal subjects [6], [7]. Second, HNE exerts various deleterious effects on lung immunity and structure such as degradation of surfactant proteins and induction of emphysema [8]–[10].

Because airway epithelial cells represent the first line of lung responses to invading bacteria, we sought to determine if HNE impairs airway epithelial host defense functions. In the current study, we focused on the effects of HNE on the function of a recently described host defense protein short palate, lung, and nasal epithelium clone 1 (SPLUNC1). A previous study suggests that HNE is involved in SPLUNC1 degradation [11]. However, the role of HNE-mediated SPLUNC1 degradation in host defense against bacterial infection has not been investigated.

SPLUNC1 is produced by large airway epithelial cells in humans and mice [12]–[14]. Human and mouse SPLUNC1 proteins at their precursor (non-secreted) forms have 256 and 278 amino acids (AA), respectively. When secreted, the N-terminal 19 AA signal peptide of SPLUNC1 precursor is cleaved to form the mature protein. Based on its structural homology to bactericidal permeability-increasing protein (BPI, a protein mainly from neutrophil granules) [15], SPLUNC1 has been predicted to exert an antimicrobial activity [16], [17]. Our published data suggest that SPLUNC1 inhibited the growth of *Mycoplasma pneumoniae* (Mp) [18], a bacterium involved in asthma as well as COPD. Using our recently generated SPLUNC1^−/−^ mice, we further confirmed the *in vivo* host defense function of SPLUNC1 during Mp infection [19]. Reduced SPLUNC1 protein has been reported in nasal lavage fluid of “healthy” human smokers [20], [21]. Moreover, SPLUNC1 expression is lower in brushed bronchial epithelial cells from “healthy” smokers than healthy non-smokers [22].

We hypothesized that HNE impairs human lung defense functions by decreasing airway epithelial SPLUNC1 levels. Our current study demonstrates that: (1) HNE directly reduced human airway epithelial defense against NTHi; (2) HNE degraded human SPLUNC1 protein; (3) Recombinant SPLUNC1 protein reduced NTHi levels in HNE-treated human airway epithelial cells; (4) NTHi levels in lungs of SPLUNC1 knockout mice were increased compared to wild-type mice; and (5) SPLUNC1 was reduced in lungs of COPD patients. Together, our findings provide a novel mechanism underlying bacterial infections in COPD patients, and a potential therapy to more effectively treat AECOPD.

## Methods

### Ethics Statement

Experimental animals used in this study were covered by a protocol approved by Institutional Animal Care and Use Committee (IACUC) of National Jewish Health, Denver, Colorado, USA. All experimental procedures were carried out to minimize animal discomfort, distress, and pain by following the American Veterinary Medical Association Guidelines. All human materials such as bronchoalveolar lavage used in this study were approved by Institutional Review Board (IRB) of National Jewish Health, Denver, Colorado, USA. The written informed consent was waived by National Jewish Health IRB for de-identified organ donors without lung diseases from whom primary normal human tracheobronchial epithelial cells were obtained through the International Institute for the Advancement of Medicine (IIAM) (http://www.iiam.org).

### Generation of Recombinant Human SPLUNC1 Protein

Recombinant human SPLUNC1 protein was generated as we previously reported [23], [24].

### Incubation of Recombinant Human SPLUNC1 Protein with Human Neutrophil Elastase (HNE)

HNE was purchased from the Elastin Product Company (Pacific, MO), and diluted in 0.05 M sodium acetate solution with 0.1 M NaCl (pH 5.0). SPLUNC1 protein at 10 µg/ml, a physiologic dose as we reported [23], was incubated with HNE (1 µg/ml [or 0.04 U, enzymatic activity] to 50 µg/ml [2.18 U], a dose range reflecting COPD lungs [25]) or 0.05 M sodium acetate solution (vehicle, control) in 96-well plates at 37°C for 30 minutes. At the end of incubation, SPLUNC1 protein was analyzed by Western blot.

### HNE Treatment in Cultured Well-differentiated Primary Human Airway Epithelial Cells

Primary normal human tracheobronchial epithelial cells were obtained from tracheas and bronchi of deidentified organ donors by digesting the tissue with 0.2% protease solution and then subjected to air-liquid interface (ALI) cultures, as we reported [26]. On day 6 of ALI culture, antibiotic gentamicin was removed from both apical surface and basolateral side. On day 10 of ALI culture, cells were treated at the apical surface with HNE (1 to 50 µg/ml) or vehicle solution. At indicated time points, apical supernatants were collected to measure SPLUNC1 levels by using an ELISA [18], [23].

### NTHi Infection in Cultured Well-differentiated Primary Human Airway Epithelial Cells

Nontypeable *Haemophilus influenzae* (NTHi) strain 12 (kindly provided by Dr. Stephen J. Barenkamp, Saint Louis University School of Medicine, Saint Louis, MO) was plated on chocolate agar plates and incubated overnight at 37°C in 5% CO2. A single colony was used to inoculate 10 ml of brain heart infusion broth (Remel, Lenexa, KS), and allowed to grow overnight. The subculture was then washed twice with PBS and the optical density (O.D.) at a wavelength of 620 nm was determined to obtain the colony-forming unit (CFU) [27].

To determine if HNE treatment impairs epithelial defense against NTHi, cells on day 10 of ALI culture were treated at the apical surface with 50 µl of HNE (10 µg/ml) in the presence or absence of NTHi at 10^3^ CFU/transwell [28], [29]. After 48 hrs of infection, apical supernatants were collected to measure NTHi levels. To quantify cell-associated NTHi, epithelial cells were dissociated by incubation with collagenase I. Briefly, cells on the transwell membrane were treated at 37°C for 2 hrs with collagenase I (0.25 mg/ml) supplemented with DNase I (10 µg/ml), EDTA (2 mM) and DTT (0.5 mg/ml). Thereafter, cells were scraped and transferred into a 1.5 ml tube for incubation with the collagenase solution for additional 3 hrs at 37°C on a rocker (225 rpm) to further dissociate the cells. Thereafter, cells were spun down to remove the collagenase solution, followed by adding the trypsin/EDTA solution for incubation at 37°C for 5 min. After removal of trypsin/EDTA solution, cell pellets were resuspended in PBS and plated on chocolate agar plates to quantify NTHi.

To test if SPLUNC1 reduction in HNE-treated cells is responsible for increased NTHi levels, recombinant human SPLUNC1 protein (10 µg/ml) or control protein from HEK293 cells was added to the apical surface of epithelial cells after 2 hrs of HNE treatment and NTHi infection. After 46 hrs of SPLUNC1 treatment or after 48 hrs of NTHi infection, apical supernatants and cells were harvested for NTHi quantification.

### NTHi Infection in SPLUNC1 Knockout Mice

SPLUNC1 knockout (KO) mouse model was used to reveal the *in vivo* function of SPLUNC1 against NTHi. SPLUNC1 KO mice on the C57BL/6 background were generated in our laboratory and bred under pathogen-free housing conditions as previously reported [19]. SPLUNC1 KO and wild-type control mice were intranasally inoculated with NTHi (10^5^ colony forming unit [CFU]/mouse in 50 µl saline), and sacrificed after 24 hrs of infection to obtain the lung tissue for NTHi quantification. Briefly, lung tissues were homogenized and plated on chocolate agar paltes to count NTHi CFUs. All the experimental protocols were approved by Institutional Animal Care and Use Committee (IACUC) at National Jewish Health.

### Measurement of SPLUNC1 in COPD Lungs

We obtained bronchoalveolar lavage (BAL) fluid from healthy non-smokers and smokers, as well as smokers with moderate to severe COPD patients (Table 1) to measure SPLUNC1 protein by using a SPLUNC1 ELISA [18], [23]. Our research protocol was approved by the Institutional Review Board (IRB) at National Jewish Health.

**Table 1:** Characteristics of human subjects.

Subjects	N	Age	Sex (M/F)	Smoking (pack-years)	FEV_1_, % predicted	FVC, % predicted	FEV_1_/FVC%
**HNS**	5	33.0±4.2	3/2	0	86.5±0.5	84.0±2.0	84.0±0.1
**HS**	9	50.9±1.1	7/2	34.5±3.6	99.1±4.2	94.8±4.4	83.0±1.8
**COPD**	4	63.5±4.1	2/2	49.6±13.9	67.5±8.4	79.8±6.0	63.8±5.3

**HNS** = Healthy non-smokers; **HS** = Healthy smokers; **COPD** = Chronic obstructive pulmonary diseases; **FEV_1_** =  forced expiratory volume in the 1^st^ second; **FVC** = forced vital capacity.

### Statistical Analyses

One-way analysis of variance (ANOVA) was used for multiple comparisons, and a Turkey’s post hoc test was applied where appropriate. *P*<0.05 was considered significant.

## Results

### HNE Degrades Recombinant SPLUNC1 Protein

**Figure 1:**
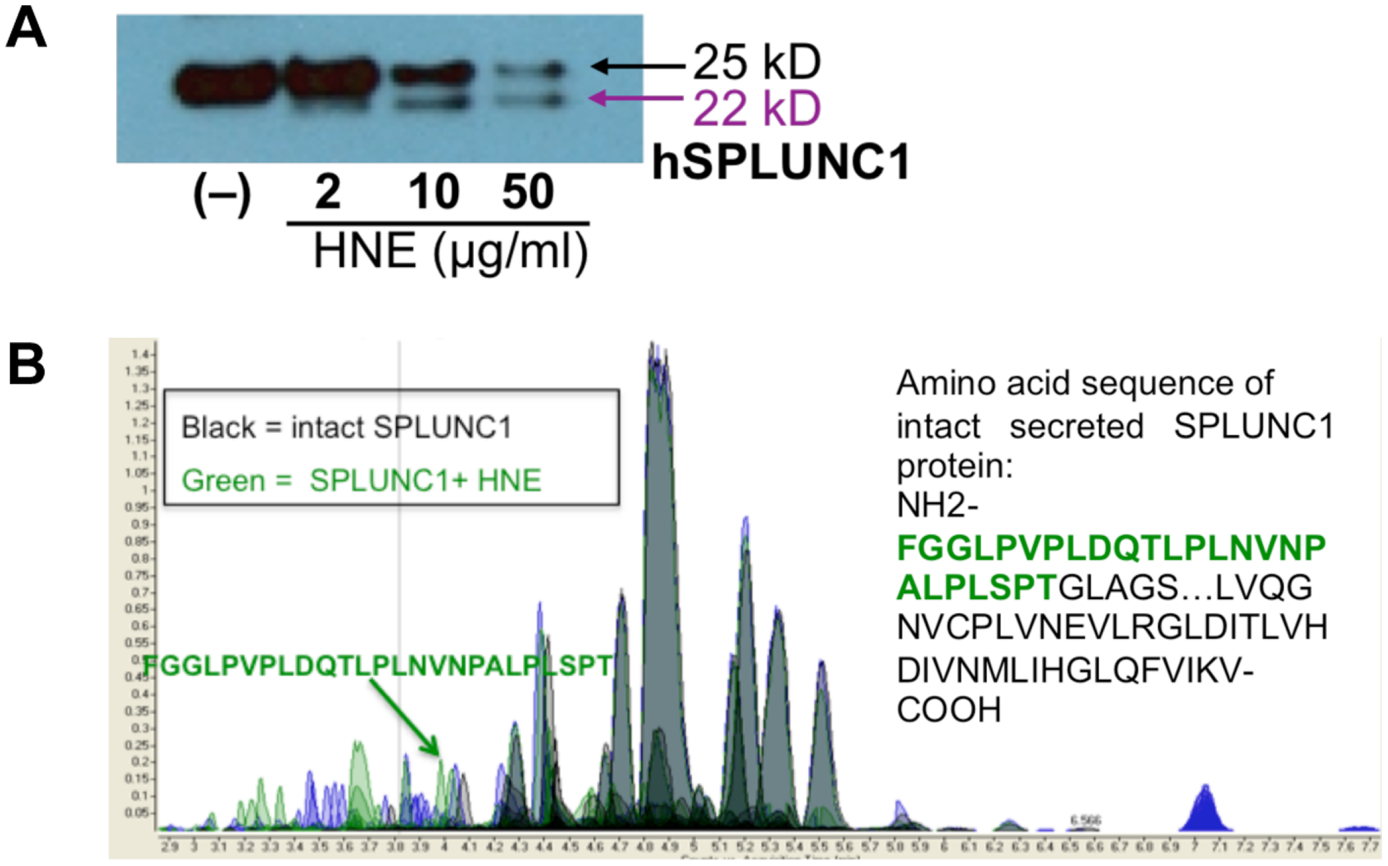
Degradation of SPLUNC1 protein by human neutrophil elastase (HNE). (**A**) Western blot of recombinant human SPLUNC1 protein (10 µg/ml) that was incubated with HNE for 30 minutes. HNE treatment dose-dependently reduces the native SPLUNC1 protein (25 kD). A fragment of 22-kD SPLUNC1 was seen in HNE-treated samples. (**B**) Mass spectrometry analysis of NHE-treated recombinant human SPLUNC1 protein revealed a 25 amino acid peptide F21-T45 (FGGLPVPLDQTLPLNVNPALPLSPT).

To test if HNE directly degrades SPLUNC1, recombinant human SPLUNC1 protein at a physiological dose (10 µg/ml) was incubated with HNE at various doses for 30 minutes. Western blot analysis demonstrated that HNE treatment significantly reduced intact SPLUNC1 (25-kD), and resulted in the formation of a 22-kD SPLUNC1 fragment (loss of a 3-kD peptide) (Figure 1A). We then used liquid chromatography mass spectrometry (LC/MS) on a quadrupole time-of-flight (QTOF) mass spectrometer to identify the 3-kD peptide. As shown in Figure 1B, a 25 amino acid peptide (F21-T45, total mass: about 3-kD) at the N-terminus of SPLUNC1 protein was identified.

### HNE Degrades SPLUNC1 Protein in Cultured Well-differentiated Human Airway Epithelial Cells

**Figure 2:**
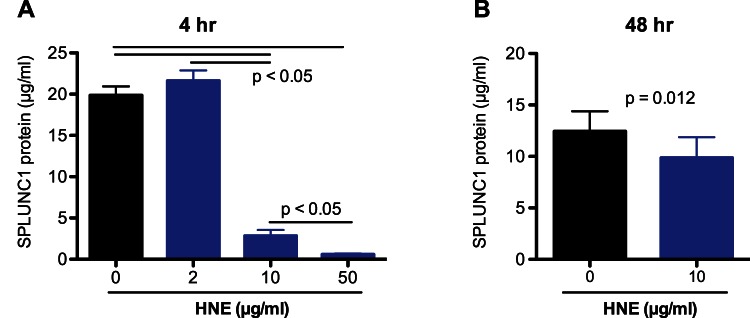
Human neutrophil elastase (HNE) decreases SPLUNC1 protein in cultured human airway epithelial cells. Primary normal human tracheobronchial epithelial cells (N = 3) were differentiated into the mucociliary phenotype under air-liquid interface culture for 10 days. After 4 (**A**) and 48 (**B**) hrs of HNE treatment, SPLUNC1 protein in apical supernatants was measured by an ELISA. Data are expressed as means ± SEM.

Having shown the ability of HNE to degrade recombinant SPLUNC1 protein in a cell-free environment, we tested if HNE reduces SPLUNC1 protein secreted from primary normal human airway epithelial cells that were well differentiated under air-liquid interface culture. HNE dose-dependently decreased SPLUNC1 after 4 hrs of HNE treatment (Figure 2A). HNE effects on SPLUNC1 were sustained even after 48 hrs of HNE treatment (Figure 2B).

### HNE Dose-dependently Increases Bacterial Load in Well-differentiated Human Airway Epithelial Cells

**Figure 3:**
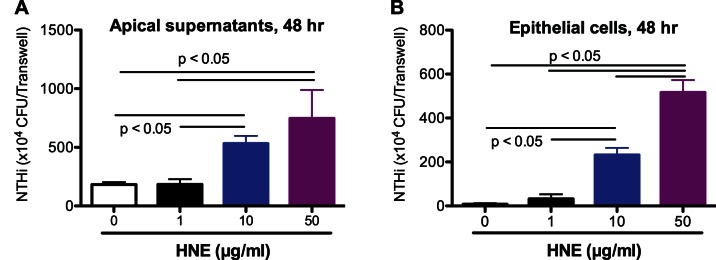
Human neutrophil elastase (HNE) dose-dependently increases the load of nontypeable *Haemophilus influenzae* (NTHi) in cultured human airway epithelial cells. Primary normal human tracheobronchial epithelial cells (N = 3) were differentiated into the mucociliary phenotype under air-liquid interface culture for 10 days, and then infected with NTHi for 48 hrs. (A) – NTHi in apical supernatants; (B) – NTHi associated with epithelial cells. Data are expressed as means ± SEM.

To determine the effects of HNE on bacterial load, apical surface of cultured well-differentiated normal human airway epithelial cells was infected with NTHi for 48 hrs in the presence or absence of HNE. NTHi levels in the apical supernatants (Figure 3A) and cells (Figure 3B) were markedly increased in a HNE dose-dependent manner.

### Recombinant Human SPLUNC1 Protein Reduces NTHi Load in HNE-treated Human Airway Epithelial Cells

**Figure 4:**
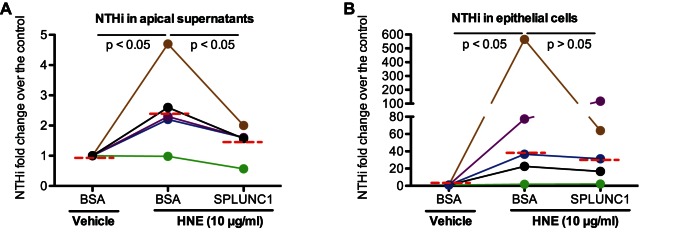
Recombinant human SPLUNC1 protein significantly reduces the load of nontypeable *Haemophilus influenzae* (NTHi) in apical supernatants of human neutrophil elastase (HNE)-treated human airway epithelial cells. Primary normal human tracheobronchial epithelial cells (N = 5) were differentiated into the mucociliary phenotype under air-liquid interface culture for 10 days, and then infected with NTHi (10^3^ CFU/transwell) in the presence or absence of HNE (10 µg/ml). After 2 hrs of NTHi and HNE treatment, recombinant human SPLUNC1 protein (10 µg/ml) was added to the apical surface of epithelial cells. After 46 hrs of SPLUNC1 treatment or after 48 hrs of NTHi infection, apical supernatants and cells were harvested for NTHi quantification. (A) – NTHi in apical supernatants; (B) – NTHi associated with epithelial cells. Each connected color line represents the data collected from cells of an individual donor. The horizontal dotted red lines indicate medians of NTHi fold changes (HNE treatment versus vehicle solution control).

**Figure 5:**
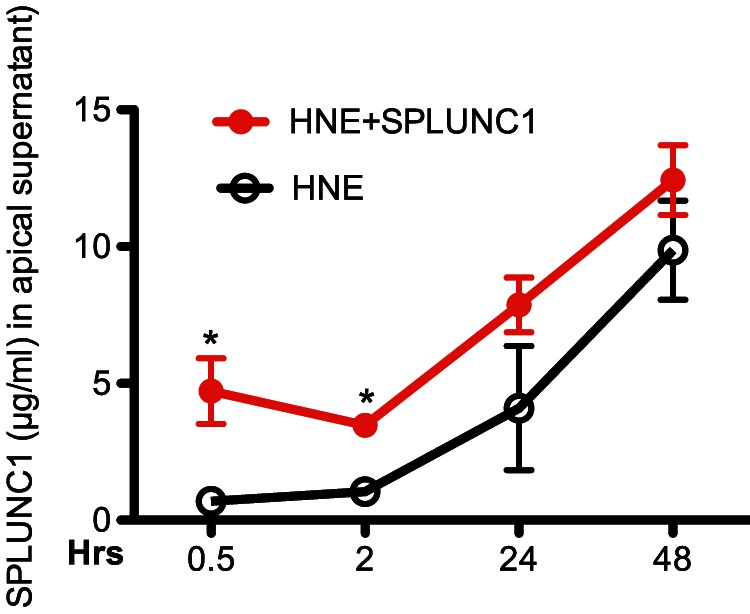
Time course study of the rescuing effects of recombinant human SPLUNC1 protein on SPLUNC1 protein levels in human neutrophil elastase (HNE)-treated human airway epithelial cells. Primary normal human tracheobronchial epithelial cells (N = 3) were differentiated into the mucociliary phenotype under air-liquid interface culture for 10 days, and then treated with HNE (10 µg/ml) in the presence or absence of recombinant human SPLUNC1 protein (10 µg/ml). At indicated time points, SPLUNC1 protein in the apical supernatants was measured by using an ELISA. Data are expressed as means ± SEM.

To determine if HNE-mediated SPLUNC1 reduction is responsible for impaired airway epithelial defense against NTHi (e.g., increased bacterial load), recombinant human SPLUNC1 protein (10 µg/ml) was added 2 hrs after HNE treatment and NTHi infection. After 48 hrs of infection, NTHi levels were measured in apical supernatants and cells. First, in line with data shown in Figure 3, HNE increased NTHi load in both apical supernatants and epithelial cells. Second, recombinant SPLUNC1 protein consistently reduced NTHi (43% reduction, p<0.05) in apical supernatants (Figure 4A) of epithelial cells obtained from 5 different donors. Although bacterial levels in HNE-treated epithelial cells were slightly reduced by SPLUNC1, this reduction was not statistically significant (Figure 4B). Third, we performed a time course study to observe the rescuing effect of recombinant SPLUNC1 protein on HNE-mediated SPLUNC1 reduction. As shown in Figure 5, in HNE-treated cells, recombinant SPLUNC1 protein increased SPLUNC1 levels at the apical surface for the entire 48 hrs, particularly during the first 2 hrs.

### Increased Lung NTHi Load in SPLUNC1 Knockout Mice

**Figure 6:**
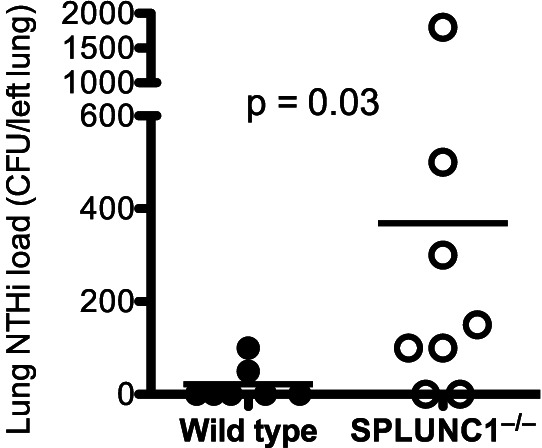
SPLUNC1 knockout (SPLUNC1^−/−^) mice increase the load of nontypeable *Haemophilus influenzae* (NTHi) in the lung. SPLUNC1^−/−^ and wild-type mice (n = 7 or 8 mice/group) were intranasally inoculated with NTHi (10^5^ CFUs/mouse). After 24 hrs of infection, left lungs were homogenized to quantify NTHi load by culture. The horizontal lines represent means of CFUs.

We have shown that SPLUNC1 in cultured human airway epithelial cells is critical to NTHi defense, but the *in vivo* function of SPLUNC1 during NTHi infection is unclear. We utilized our SPLUNC1 knockout (SPLUNC1^−/−^) mouse model to delineate the *in vivo* function of SPLUNC1. After 24 hrs of an intranasal inoculation of NTHi, bacterial load in SPLUNC1^−/−^ mice was about 30-fold higher than that in wild-type mice (Figure 6).

### Reduced SPLUNC1 in Human COPD Lungs

**Figure 7:**
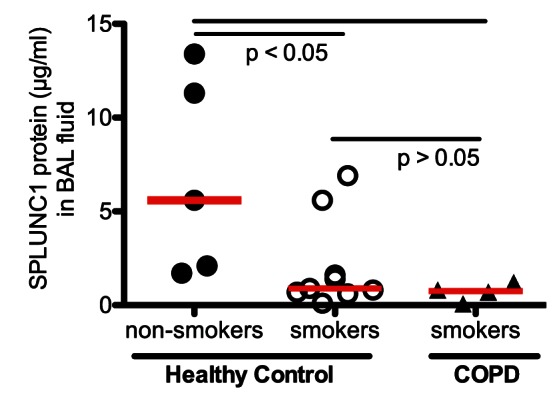
SPLUNC1 protein is decreased in the lungs of patients with chronic obstructive pulmonary disease (COPD). SPLUNC1 protein was measured in bronchoalveolar lavage (BAL) fluid of healthy non-smokers (n = 5), healthy smokers (n = 9) and COPD smokers (n = 4). The horizontal solid red lines indicate medians.

We measured SPLUNC1 protein levels in BAL fluid of healthy non-smokers, healthy smokers and stable moderate to severe COPD patients. As shown in Figure 7, SPLUNC1 protein was significantly reduced in smokers with or without COPD as compared to healthy non-smokers.

## Discussion

The current study demonstrates that human neutrophil elastase (HNE)-mediated SPLUNC1 degradation impairs airway epithelial defense against bacterial infection. We found that HNE markedly reduces SPLUNC1 in well-differentiated human airway epithelial cells and subsequently increases bacterial load, which can be attenuated by recombinant human SPLUNC1 protein. Moreover, we found decreased SPLUNC1 in human COPD lungs, and increased lung bacterial load in SPLUNC1 knockout mice. Together, our data suggest that increased HNE, a feature of excessive inflammation during acute exacerbations of COPD and other chronic lung diseases, may contribute to bacterial infections in part through degrading host defense protein SPLUNC1.

Acute exacerbations of COPD (AECOPD) pose the highest risk (i.e., mortality) to patients and the greatest costs for COPD-related healthcare. Respiratory bacterial and viral infections are the major cause of AECOPD, which commonly recruit neutrophils into the airway lumen and activate them. During neutrophil activation, they release various mediators including HNE. Although the NE knockout mouse model suggests a beneficial role of intracellular NE in host defense against bacterial (e.g., *Pseudomonas aeruginosa*) infection [30], the impact of released HNE on lung (airway) bacterial infections remains poorly understood. We have found increased bacterial (e.g., NTHi) load in HNE-treated well-differentiated primary human airway epithelial cells. Our data suggest a new mechanism underlying frequent bacterial infection during AECOPD that is characterized by excessive lung inflammation especially neutrophils.

How HNE impairs airway epithelial defense against NTHi is unclear. Our current study focuses on the effects of HNE on SPLUNC1 as recent studies suggest that SPLUNC1 exerts both *in vitro* and *in vivo* host defense functions [18], [19], [31]. First, we demonstrated that in a cell-free environment, HNE dose-dependently reduced SPLUNC1 levels. By Western blot analysis, we identified a 22-kD SPLUNC1 fragment resulting from HNE treatment, suggesting a loss of 3-kD peptide from the 25 kD native SPLUNC1 protein. By using mass spectrometry, we identified a 25 amino acid peptide (mass: ∼3 kD), suggesting a cutting site by HNE. Whether other sites of SPLUNC1 protein can be cut by HNE remains to be explored. Second, we confirmed that HNE also reduced SPLUNC1 levels in cultured well-differentiated primary normal human airway epithelial cells. Interestingly, the differences of SPLUNC1 levels between HNE-treated and control (no HNE) cells were smaller at 48 hr than at 4 hr, which may be explained by several factors, including continuous secretion of SPLUNC1 protein from epithelial cells, the loss of HNE activity over time and secretion of protease inhibitors from epithelial cells. Collectively, our data indicate that HNE can degrade SPLUNC1 secreted from airway epithelial cells under a neutrophilic inflammatory setting.

One of the major research questions is whether HNE-mediated SPLUNC1 reduction/degradation in airway epithelial cells is responsible for increased bacterial load following HNE treatment. To address this, we tested the therapeutic effects of recombinant human SPLUNC1 protein on NTHi load in HNE-treated cells. We confirmed that SPLUNC1 protein was able to restore epithelial defense functions by decreasing NTHi levels. Moreover, we demonstrated a critical *in vivo* role of SPLUNC1 in lung defense against NTHi by using the SPLUNC1 knockout mouse model. Together, our data suggest that SPLUNC1 protein may serve as a novel approach to treat bacterial infections in various lung diseases characterized by excessive neutrophilic inflammation. Although recombinant SPLUNC1 protein reduced bacterial load in HNE-treated airway epithelial cell, it did not reduce the bacteria to the normal level. This suggests that HNE may use other mechanisms to increase bacterial load. Indeed, a previous study demonstrated that HNE increased epithelial permeability [32]. Therefore, it is possible that HNE could increase bacterial binding in part through disrupting epithelial barrier function in our cell culture model. The contribution of HNE-mediated SPLUNC1 reduction versus epithelial barrier disruption to increased bacterial load will be investigated in our future studies.

Although the current study was focused on *in vitro* studies, we examined SPLUNC1 levels in COPD lungs. We found reduced SPLUNC1 levels in COPD patients as compared to healthy controls, especially healthy non-smokers. Interestingly, SPLUNC1 levels in healthy smokers were less than those in healthy non-smokers. We realize that SPLUNC1 reduction *in vivo* may be caused by multiple factors. We measured HNE levels in BAL fluid of COPD patients who were stable during the BAL procedure and control subjects, and did not find a significant difference of HNE. This may be explained by the fact that our patients undergoing bronchoscopy and BAL fluid procedure were stable (no acute exacerbations). Therefore, SPLUNC1 reduction in stable COPD patients may be caused by other factors such as cigarette smoke exposure. In a pilot study, we exposed well-differentiated human airway epithelial cells to whole cigarette smoke or air (control) for 10 minutes as we described before [33]. After 24 hrs of cigarette smoke exposure, SPLUNC1 levels in the apical surface were reduced by 40%. Future studies should correlate SPLUNC1 levels with HNE and bacterial load in COPD patients before, during and after acute exacerbations. This could be done by obtaining sputum samples, as sputum collection is safer than the bronchoscopy procedure for patients during AECOPD.

Our current study has several limitations. First, we only used normal human airway epithelial cells to study the impact of HNE on SPLUNC1 degradation and bacterial infection. We will consider COPD airway epithelial cells in our future studies to determine if COPD cells may differ from normal cells in their responses to HNE and NTHi infection. Second, since NTHi was the only strain of bacteria examined in the current study, our results should be verified in other strains of bacteria involved in COPD, including *Pseudomonas aeruginosa*. Third, HNE may degrade other antimicrobial substances (e.g., surfactant protein A, [8]) that promote host defense against bacteria. The relative contribution of HNE-mediated reduction of SPLUNC1 versus other antimicrobial mediators should be dissected in future studies. Lastly, we realize the variability of bacterial load data in human airway epithelial cells from different donors. This may be caused by the variation in susceptibility of different donor cells to bacterial infection. More experiments from additional donors may be needed to address the underlying mechanisms of NTHi load variation. Furthermore, *in vivo* (e.g., mouse) studies are warranted to determine the physiological significance of a less than one log bacterial load change in cultured well-differentiated human primary airway epithelial cells treated with recombinant SPLUNC1 protein and/or HNE.

In summary, our current study provides a new mechanism to explain increased susceptibility of COPD patients to bacterial infections, in particular during acute exacerbations that are featured with excessive neutrophilic inflammation and release of neutrophil elastase. As SPLUNC1 is an endogenous protein and unlikely has the side effects (e.g., drug resistance) of antibiotics, it has the potential to treat bacterial infections associated with AECOPD and perhaps other lung diseases such as asthma and cystic fibrosis.
